# tRNA Synthetases Are Recruited to Yeast Ribosomes by rRNA Expansion Segment 7L but Do Not Require Association for Functionality

**DOI:** 10.3390/ncrna7040073

**Published:** 2021-11-22

**Authors:** Nina Krauer, Robert Rauscher, Norbert Polacek

**Affiliations:** Department of Chemistry, Biochemistry and Pharmaceutical Sciences, University of Bern, 3012 Bern, Switzerland; nina.krauer@bluewin.ch

**Keywords:** ribosome biology, rRNA expansion segments, aminoacyl-tRNA synthetases, translation regulation

## Abstract

Protein biosynthesis is essential for any organism, yet how this process is regulated is not fully understood at the molecular level. During evolution, ribosomal RNA expanded in specific regions, referred to as rRNA expansion segments (ES). First functional roles of these expansions have only recently been discovered. Here we address the role of ES7L_a_ located in the large ribosomal subunit for factor recruitment to the yeast ribosome and the potential consequences for translation. Truncation of ES7L_a_ has only minor effects on ribosome biogenesis, translation efficiency and cell doubling. Using yeast rRNA deletion strains coupled with ribosome-specific mass spectrometry we analyzed the interactome of ribosomes lacking ES7L_a_. Three aminoacyl-tRNA synthetases showed reduced ribosome association. Synthetase activities however remained unaltered suggesting that the pool of aminoacylated tRNAs is unaffected by the ES deletion. These results demonstrated that aminoacylation activities of tRNA synthetases *per se* do not rely on ribosome association. These findings suggest a role of ribosome-associated aminoacyl-tRNA synthetase beyond their core enzymatic functions.

## 1. Introduction

Protein biosynthesis is essential for life and belongs to the most central and probably most ancient biochemical processes that exist. This fact is mirrored in the machinery performing the task of sequential addition of amino acids to a growing nascent chain, translation. It probably evolved before the last universal common ancestor about 4 billion years ago [1]. The active sites of the central enzyme of protein biosynthesis (the ribosome) remained virtually unaltered during the evolution across all kingdoms [2]. Nevertheless, ribosomes dramatically expanded during the course of evolution in regions not directly involved in the basic steps of translation (e.g., codon recognition, peptide bond formation, translocation) [3,4]. Comparing bacterial and eukaryotic ribosomes, the number of ribosomal proteins increased from 54 (in *E. coli*) to 79 (in *S. cerevisiae*). Furthermore, the ribosomal RNA (rRNA) expanded in length and complexity. Of special interest are substantial expansions of specific rRNA helices ranging from 7–210 nucleotides in the budding yeast *S. cerevisiae* up to expansions of several hundred nucleotides in mammalian rRNAs [5]. Some of these so-called expansion segments (ES) have established roles in ribosome biogenesis [5] and inter-subunit bridge formation and all of them are located at the periphery of the ribosomal subunits and face towards the cytosol [6]. Others evolved as recruitment platforms for cotranslationally acting factors, such as the Met-aminopeptidase complex [7,8] or mRNAs [9]. Many other ES however are poorly studied and their function at the translating ribosome remains elusive. 

The largest ES in yeast is ES7L located at the back of the large ribosomal subunit. In *S. cerevisiae* it contains three arms of different length which are further expanded in mammalian ribosomes. Early research established a crucial role in ribosome biogenesis; complete deletion of ES7L is lethal due to the lack of mature 60 S ribosomal subunits [5]. ES7L further serves as a site of regulatory cleavage as part of a stress response process that removes damaged ribosomes from the pool of actively translating ones [10,11,12]. Recent studies furthermore suggest a role of ES7L in factor recruitment to eukaryotic ribosomes, more precisely, aminoacyl-tRNA synthetases (AARS) [13]. 

AARS which are associated to the ribosome have been described to engage translation factors to efficiently recharge elongation factor 1 with a charged tRNA [14,15]. Fast recycling of tRNAs would increase the local concentration of aminoacylated tRNAs thus boosting translation efficiency [16]. Similar mechanisms have been described in archaea, suggesting AARS recruitment to ribosomes as a more general strategy to enhance local recharging of tRNAs [17,18]. In eukaryotes, several AARS assemble into complexes (multisynthetase complex (MSC)) of varying degrees of complexity [19]. The mammalian MSC was shown to associate to translated mRNAs in human and hamster cells in order to boost local translation [20,21,22]. The MSC was further found to bind the actin cytoskeleton, which links translation to cellular plasticity but also suggest that regulation of translation differs depending on cellular subenvironments [21]. In yeast, association of methionyl-tRNA synthetase (MetRS) and Arc1p in the MSC increases substrate affinity and efficiency of the aminoacylation reaction [23]. The mammalian MSC, however, rather appears to function as a containment unit that does not enhance or alter the genuine function of AARS but rather regulates their cellular location [20,24]. The question therefore stands which relevance the association of AARS into complexes has for tRNA aminoacylation and consequently for translation.

In this study we investigated the general role of ES7L_a_ in yeast ribosome biology and more specifically in factor recruitment during translation. The data presented here suggests that ES7L_a_ plays a role in the recruitment of AARS to the ribosome. However, this ribosome-association of AARS is dispensable for tRNA charging efficiency and delivery to the ribosome. 

## 2. Materials and Methods

### 2.1. Yeast Strains and Cultivation

The desired strains were produced by plasmid shuffling in the KAY488 strain which lacks the entire RDN1 gene and relies on a plasmid (pRDN-hyg carrying a URA3 marker) as rRNA source. The target plasmid (pNOY 373, containing a Leu2 selection marker) carrying the desired mutations within the rDNA was transformed into cells and selected for by FOA treatment. Selected cells were tested for correct rRNA sequences by sequencing and poisoned primer extension. Yeast cells were grown in minimal medium lacking the amino acids leucine (SC-medium) at 30 °C and 220 rpm shaking. Cells were grown for at least 24 h in liquid culture before experiments were conducted.

To analyze cell growth, cells were diluted to OD_600_ = 0.1 in fresh, prewarmed SC-medium and grown for 4 h. 400 mL cell suspension was transferred to a 48-well plate and incubated for 24 h in a Tecan plate reader (30 °C, 196 rpm, 4 mm shaking amplitude). Cell density was measured every 15 min. Growth rates were calculated from the exponential phase. In parallel, cells were inoculated to 50 mL liquid cultures and cell density was measured in 1 h intervals. 

### 2.2. Polysomes

Cells were grown to OD_600_ = 0.8 in 200 mL SC-medium, filtered and snap frozen. Material was ground in a cryomill in the presence of polysome buffer (10 mM Tris/HCl, pH 7.4, NaCl, Triton X-100, MgCl_2_, 5 mM PMSF, 2 mM DTT, 100 mg/mL Cycloheximide). Powders derived from 200 mL cultures were mixed with 500 mL polysome buffer and thawed at 30 °C. Lysates were cleared and directly loaded on a Sepharose-4b column for size exclusion. The column was washed, equilibrated and eluted with polysome buffer. Fractions containing polysomes were directly loaded on a 10–40% sucrose gradient in polysome buffer and ultracentrifuged (25,000 rpm, 5 h, 4 °C, SW32Ti Beckman Coulter, Brea, CA, USA). Gradients were pumped out and fraction containing the polysomes were collected and pelleted by centrifugation (39,000 rpm, 16 h, 4 °C, SW41Ti Beckman Coulter, Brea, CA, USA). Ribosome pellets were snap frozen and served as input material for mass spectrometric shotgun protein identification/quantification. Mass Spectrometry analysis was performed at the Proteomics & Mass Spectrometry Core Facility (PMSCF) of the University of Bern. Trypsinized peptides were analyzed by LC-MS with a Fusion Lumos ETD connected to a nano-UPLC column. The peptide intensities were quantified by the MaxQuant LFQ algorithm [25]. The fold changes were calculated as ratios of ΔES7L_a_ over wild-type levels and log2-transformed to obtain log2-fold changes. GO-Term analysis was performed using the DAVID data base [26,27]. Whole cell lysates were TCA precipitated and served as a control for ribosome samples prepared as described above. Similarities between samples were assessed using principal component analysis. Here, we used the prcomp function embedded into the stats package in R. Quantified protein levels served as input data for assessment of deviation in between samples.

### 2.3. Western Blot

Fractions obtained from size exclusion chromatography as described above where precipitated in 10% TCA on ice and pelleted by centrifugation (14,000× *g*, 10 min, 4 °C). Pellets were washed twice in 100% ice-cold acetone and dried. Proteins were resuspended in SDS loading dye and loaded on a 10–12% SDS-polyacrylamide gel. Samples were separated, transferred to nitrocellulose membranes and detected using anti-HA (12CA5, mouse IgG, Merck, Kenilworth, NJ, USA) and anti-RPS6 (ab40820, rabbit IgG, Abcam, Cambridge, UK) antibodies.

### 2.4. Metabolic Labeling

Cells were inoculated to OD_600_ = 0.4 and incubated for 2 h. 5 OD units were then washed twice in minimal medium lacking the amino acid methionine (SC-Met). Cell were resuspended and suspension was mixed with 4.5 mL SC-Met containing 7 mL (^35^S)-methionine (0.1 ng/mL, 10 μCi/μL, Hartmann Analytics, Braunschweig, Germany) and 0.8 mL cold Methionine (0.3125 mg/mL). Immediately, a 0 min sample (500 mL) was taken and TCA precipitated. Cells were incubated at 30 °C and 220 rpm shaking, and samples were taken after 10, 20, 30 and 60 min and precipitated. Samples were resuspended in SDS-loading buffer, boiled (95 °C, 5 min) and separated in a 10% SDS-polyacrylamide gel. Gels were stained with Coomassie, dried and exposed prior to detection of radioactivity using a Typhoon FLA 9500 (GE Healthcare, Chicago, IL, USA) phosphoimager.

### 2.5. Total RNA Isolation

Cells were grown to OD_600_ = 0.8 and harvested by centrifugation. Pellets were washed, mixed with SDS (f.c. = 1%) and hot acidic phenol was added. Samples were incubated at 65 °C for 5 min at 1200 rpm shaking. After centrifugation (14,000× *g*, 5 min), the aqueous phase was transferred to a new precooled tube on ice. An equal volume of PCI (phenol: chloroform: isoamylalcohol 25:24:1, AppliChem Panreac, Chicago, IL, USA) was added, mixed by vortexing, and centrifuged (14,000× *g*, 5 min, 4 °C). The upper phase was again transferred to a new tube, sodium acetate (3 M, pH 5.3) was added to 0.3 M and 2.5 vol. of ice-cold 100% ethanol were added, and mixed. Precipitation was performed overnight at −80 °C. The RNA was pelleted by centrifugation (14,000× *g*, >40 min, 4 °C), supernatant discarded, and the pellet washed once with ice-cold 80 % ethanol. The pellet was dried for 5 min at 37 °C and resuspended in deionized water. Isolation of charged tRNAs was performed at RT instead of 65 °C.

### 2.6. Poisoned Primer Extension

Extension assays were performed as described earlier [28]. Briefly, total RNA was reverse transcribed using radioactively labelled rRNA specific RT-primers in a setup containing _dd_TTP. The RT reaction stopped after the first adenosine in the sequence was encountered which yielded distinct products for the wild-type and mutant sequences. The RT products were separated by electrophoresis and sequencing reaction were run to confirm the sequence of interest. Gels were frozen to avoid diffusion and exposed for 72 h. Signal detection was performed using a Typhoon FLA 9500 (GE Healthcare, Chicago, IL, USA) phosphoimager.

### 2.7. tRNA Northern Blots

A denaturing polyacrylamide gel (20 × 20 cm, width: 2 mm) was pre-run for 10 min at 120 V in 1× TBE. Samples were mixed with an equal volume of 2× RNA Loading Dye + EtBr (Thermo Scientific, Waltham, MA, USA), heated up to 95 °C, 2 min and loaded on the gel after flushing the wells with a syringe. The gel was electrophoresed for 2 h at 250 V, total RNA was detected using ethidium bromide staining. RNA was transferred to a nylon membrane (Amersham Hybond-N+, GE Healthcare, Chicago, IL, USA) by semidry blotting (400 mA, 45 min), crosslinked and hybridized with a radioactively labelled probe over night at 42 °C. Primers specifically binding tRNAs of interest were labelled using [γ-^32^P]-ATP (Hartmann Analytic, Braunschweig, Germany, 0.37 MBq/μL) in a PNK catalyzed phosphorylation reaction (tRNA Proline: GAACCCAGGGCCTCTC, tRNA Methionine CAGGGGAGGTTCGAAC, tRNA Glutamine: ACCCGGATTCGAAC, tRNA Phenylalanine: GATCTTCAGTCTGGCG). Membranes were washed and dried prior to exposure and signal detection in a Typhoon FLA 9500 (GE Healthcare, Chicago, IL, USA) phosphoimager.

### 2.8. rRNA Northern Blot

rRNA northern blots were performed as described earlier [7]. 20 mg of total RNA were denatured in loading dye (95 °C, 3 min) and electrophoresed (100 V) in a 1% denaturing agarose gel for 4 h. Total RNA was detected by ethidium bromide staining. rRNA was transferred to a nylon membrane by passive transfer (24 h, RT, 20× SSC). RNA was crosslinked to the membrane by UV irradiation and rRNA species were probed with radioactively labeled probes (ITS1: GGCCAGCAATTTCAAGTTA, ITS1: CGGTTTTAATTGTCCTA). Primers were labelled using [γ-32P]-ATP (Hartmann Analytic, Braunschweig, Germany, 0.37 MBq/μL) in a PNK catalyzed phosphorylation reaction. Membranes were hybridized (overnight at 42 °C), washed and dried prior to exposure and signal detection in a Typhoon FLA 9500 (GE Healthcare, Chicago, IL, USA) phosphoimager.

## 3. Results

### 3.1. Removal of Expansion Segment ES7L_a_ Slightly Reduces Cellular Fitness and Translation Capacity

**Figure 1:**
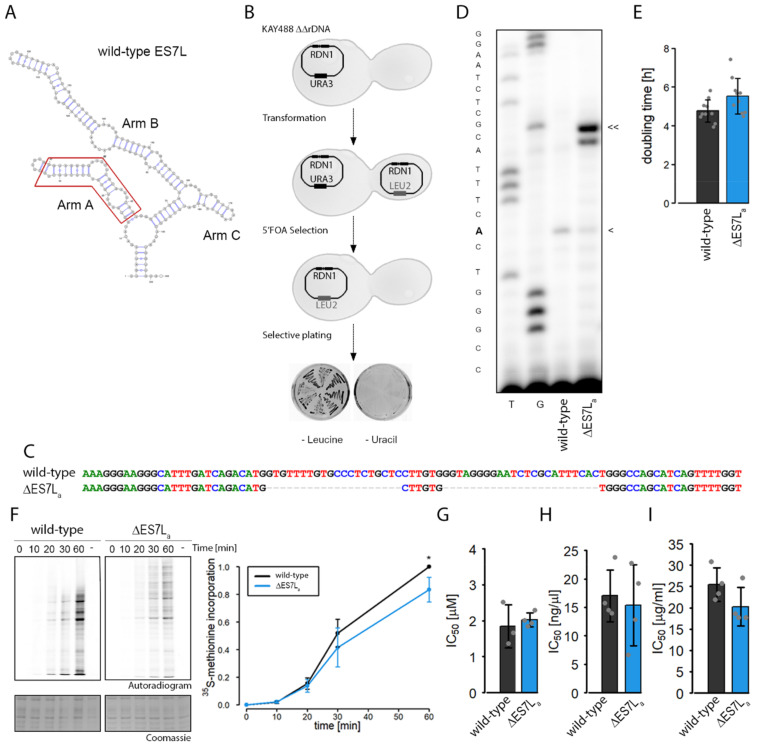
Effects of ES7L A-arm deletion on growth rate and translation. (**A**) Predicted secondary structure of ES7L. The secondary structure model was predicted using mFold [31]. Arms are indicated, and the deleted region is highlighted in a red frame. (**B**) Schematic presentation of the plasmid exchange strategy. The rDNA deletion strain KAY488 was transformed with pNOY373 encoding mutant rRNA. Strains were selected on selective plates and positive clones grew on—Leucine but not on—Uracil plates. (**C**) Plasmids were re-isolated from the positive strains and sequenced. Lines indicate gaps in the alignment which are in line with the deletion strategy. (**D**) Poisoned primer extension was performed to ensure that the strains only express mutant rRNA. The primer extension reaction stopped at the first A encountered (marked with < for wild-type and << for the deletion strain) because of addition of ddTTP to the reaction mixture. Total RNA isolated from the deletion strains served as template for primer extension. As controls, two sequencing reactions (T, G) and a primer extension with wild-type RNA were performed. (**E**) Doubling time of each strain obtained with plate reader experiments in YPD medium. (**F**) Metabolic labelling experiment was performed with cells from all four strains. Numbers depict time points after which samples were isolated. “-“ indicates a cycloheximide control. Quantified metabolic labelling results of wild-type and ∆ES7L_a_. The ^35^S-methionine signal in the autoradiogram was normalized to the signal of the Coomassie staining and finally to wild-type at timepoint 60 min (3 biological replicates). Significant 20% reduction of translation was observed after 60 min (Significant differences were determined using the two-tailed unpaired Student’s *t*-test (* *p* < 0.05). (**G**–**I**) Sensitivity to antibiotics: IC_50_ calculated from growth rates in different antibiotic concentrations (G: Anisomycin, H Cycloheximide, I: Blasticidin S). All data is indicated as mean ± standard deviation.

To understand the role of expansion segment 7L in translation regulation or factor recruitment, we removed the A-arm from the 25S rRNA (Figure 1A, as deduced from previously published structures of yeast ribosomes [29]). We successfully selected for cells expressing exclusively truncated rRNA (Figure 1B). To this end the truncated 25S rRNA gene was expressed from a plasmid in a yeast strain lacking all chromosomally encoded rDNA genes [30]. The success of this experimental strategy was confirmed by sequencing (Figure 1C) and poisoned primer extension assays (Figure 1D). Both experimental strategies confirmed the desired deletion of the A-arm of ES7L on 25S rRNA and the exclusive presence of truncated ribosomes in the strain (referred to as ∆ES7L_a_ hereafter). 

Having established the deletion strain, physiological alterations caused by the rRNA truncation were explored. The growth rates were slightly decreased for ∆ES7L_a_ cells as compared to the wild-type (Figure 1E). Since part of the rRNA was deleted we hypothesized that the reduction in cell growth was caused by reduced ribosomal activity. To test this hypothesis, metabolic labeling experiments were conducted (Figure 1F). As suggested by the mild effect on growth rates we could only observe a mild reduction of incorporation of radioactively labeled methionine into proteins (Figure 1F). Reduced translation efficiency could be conferred by a global reduction of translation competent ribosomes or by specific alterations acting on substeps of translation such as initiation, elongation or termination. To test whether substeps of elongation were less efficient in ∆ES7L_a_ ribosomes, we subjected cells to increasing concentrations of antibiotics that inhibit specific steps of protein biosynthesis. Growth rates were measured for these conditions and IC_50_ was determined. We did not observe any change in IC_50_ for any of the antibiotics tested: anisomycin (inhibits peptide bond formation), cycloheximide (inhibits translocation) or blasticidin S (inhibits termination); Figure 1G–I) suggesting that these steps are not more vulnerable in ∆ES7L_a_ cells.

**Figure 2:**
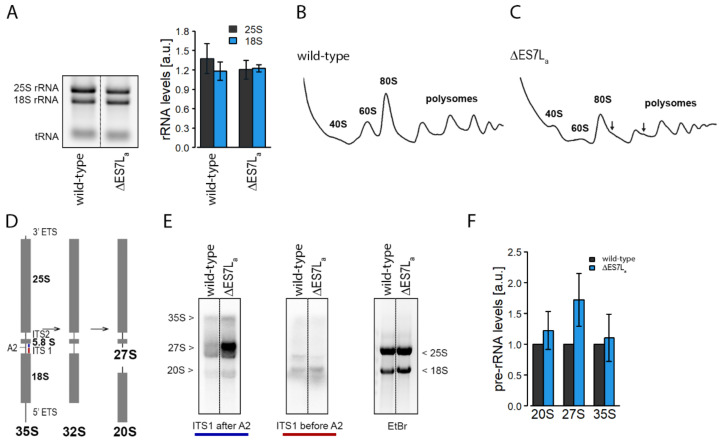
∆ES7La changes ribosome maturation and 60S levels. (**A**) Relative 18S and 25S rRNA levels (normalized to tRNA) of the different strains as deducted from gel electrophoresis of total RNA isolated from equal cell numbers (left). Mean ± standard deviation of three biological replicates is shown (right). (**B**,**C**) Polysome profiles of wild-type (**B**) and ES7L_a_ deletion strains (**C**). “Halfmer” polysomes (arrows) occur in the ∆ES7L_a_ strain. (**D**,**F**) 20S, 27S and 35S pre-rRNA were tracked by northern blot probes annealing before and after A2 cleavage within ITS1 (**D**). The bands in the northern blot (**E**) were normalized to the mature rRNA bands of the gel (EtBr) and finally to wild-type levels. The mean ± standard deviation of the relative pre-rRNA levels of three biological replicates are shown (**F**).

Earlier studies established a role of ES in ribosome biogenesis [5]. We thus investigated the cellular amount of mature rRNA as a proxy for mature ribosomes. No significantly altered rRNA levels were observed, suggesting that steady state ribosome levels were not dramatically perturbed (Figure 2A). Nevertheless, we could observe a slight reduction of 25S rRNA which did not reach statistical significance (Figure 2A). Since protein biosynthesis levels depend on the number of ribosomes associated to a given transcript, we used polysome profiling to test differential ribosome association to transcripts (Figure 2B,C). While in wild-type and ∆ES7L_a_ cells ribosomes associated similarly to polysomes only ∆ES7L_a_ polysomes displayed shoulders referred to as “halfmers” [32]. These are formed when a 40S subunit initiates translation but is not joined by a 60S subunit to form a fully assembled translation competent 80S ribosome. “Halfmers” have been described in situations of large ribosomal subunit shortage. Moreover, a mild reduction of free 60S subunit was observed which is in line with the slightly reduced levels of 25S rRNA. To learn if biogenesis of the large subunit is indeed perturbed, northern blot analyses were performed using probes that specifically detect rRNA biogenesis intermediates of the RDN1 primary transcript (Figure 2D,E). When the 20S rRNA precursor of the small subunit was investigated, no major differences in precursor accumulation were observed suggesting normal maturation of the small subunit (Figure 2E,F). In contrast, analysis of the 27S precursor revealed a profound enrichment of this biogenesis intermediate (Figure 2E,F) suggesting that indeed the large subunit lacking ES7L_a_ is ribosome biogenesis deficient. 

Taken together, this data suggests that ES7L_a_ is required for normal maturation of the large subunit and if absent creates a slight shortage of mature 60S ribosomal subunits resulting in “halfmer” formation as detected during polysome profiling and reduced overall protein synthesis.

### 3.2. ES7L_a_ Deletion Redistributes Aminoacyl-tRNA Synthetases from Ribosomes

**Figure 3:**
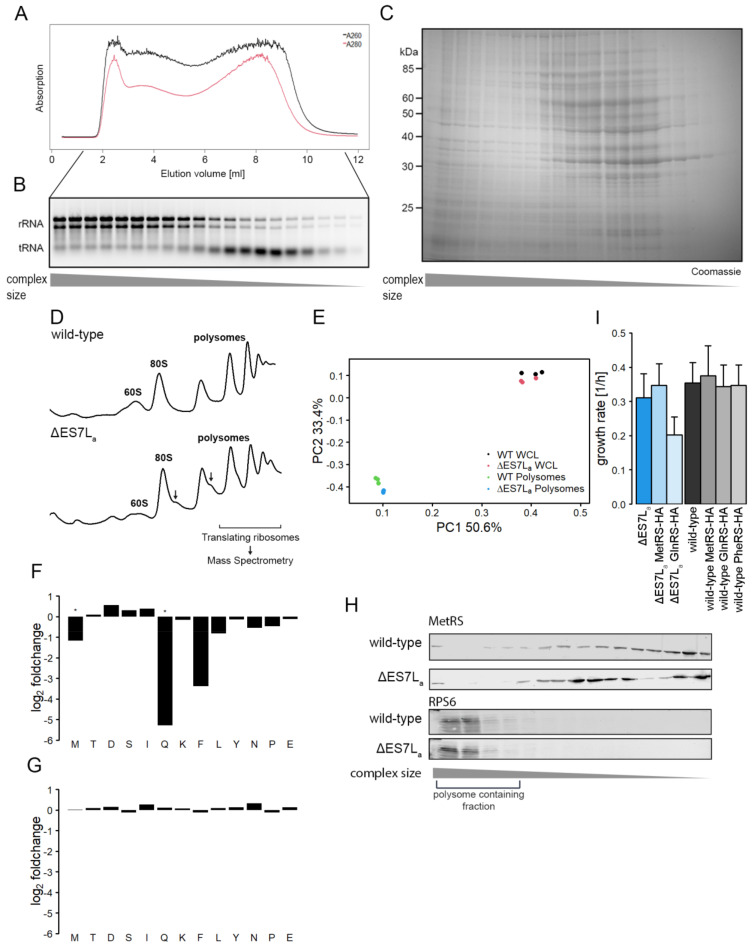
Isolation of actively translating ribosomes and identification of AARS as differential ribosome binders. (**A**) Absorption profiles of size exclusion chromatography (Sepharose 4b column, polysome buffer) as an additional purification step of translating ribosome complexes. Protein and RNA content was measured by A_260_ nm (black) and A_280_ nm (red) measurement. (**B**) Total RNA isolated from fractions of SEC in A and visualized on a 1% agarose gel. (**C**) Proteins isolated from fractions of SEC via TCA precipitation of fractions in (**A**) and visualized on a 10% SDS-PAGE. (**D**) Application of the purification method for wild-type and ∆ES7L_a_ samples to isolate translating ribosomes for MS analysis by SEC of the WCL and subsequent density gradient centrifugation. Polysome profiles are shown for one of three replicates each and “halfmer” polysomes occurring in ∆ES7L_a_ lysates are marked with an arrow. (**E**) LFQ quantified protein intensities between samples were compared using principal component analysis. Similarity between samples is indicated as closeness in the diagram. PC in axis titles depicts “principal component” (**F**) Fold changes of different detected AARSs in actively translating ribosomes, labelled with the corresponding amino acid. (**G**) Fold changes of different AARSs in the whole cell lysate, labelled with the corresponding amino acid. (*: *p*-value ≤ 0.05). (**H**) Western blot detecting HA-tagged MetRS and RPS6 as a control for ribosome association. Lysates were separated on a Sepharose 4b column, proteins were precipitated and separated on a SDS-gel. Signal was detected on a Liqor imager. (**I**) Growth rates of cells expressing genomicly tagged AARS. Data is shown as mean ± standard deviation of 6 biological replicates.

Despite the reduction of 60S ribosomal particles, the deletion strain had a very mild phenotype and displays normal amounts of translating ribosomes. To test whether ES7L_a_ plays a role in ribosome biology apart from maturation, we chose an unbiased mass spectrometric approach to quantify proteins binding to actively translating ribosomes. To decrease the amounts of contaminants, whole cell lysates (WCL) were first subjected to a Sepharose 4b size exclusion column which was described to allow isolation of actively translating ribosomes from WCL [33]. Since this procedure is not commonly used for purification of translating complexes, fractions after size exclusion chromatography were collected and total RNA and proteins extracted in order to validate the purification. A clear enrichment of rRNA in early eluting fractions (containing large complexes) as compared to later fractions was observed (Figure 3A,B), which indeed suggests the enrichment of polysomes in early fractions. tRNAs, however, were enriched in later migrating fractions. Those tRNAs engaged in translating ribosomes eluted in complex with polysomes. Free tRNAs, bound by EF1A in the cytosol, on the other hand, elute in fractions that elute later and correspond to smaller complexes. Also, the protein pattern in these fractions was strikingly different from later fractions. More specifically, early eluting fractions are devoid of several of the most abundant proteins, seen in later eluting fractions. However, small proteins which represent all the ribosomal proteins, are obtained in early fractions again indicating the purifying capacity of the approach (Figure 3C). Next, polysome profiling was performed from fractions enriched for ribosomal particles and the polysome containing fraction were used as input for mass spec experiments. WCL were used as controls. 

The polysome profiles after size exclusion showed a reduced level of free ribosomal subunits and no peak of non-ribosome associated material as seen in usual polysome profiles (Figure 3D, compare to Figure 2B,C). Again, ”halfmers” in the ∆ES7L_a_ polysomes were observed suggesting that the procedure does not perturb biological assemblies. Wild-type and ∆ES7L_a_ proteomes and ribo-proteomes showed very high global similarity (Figure 3E). A principal component analysis, which illustrates similarities in global protein levels between individual samples, revealed major differences between WCL and polysome associated proteomes (Figure 3E). Nevertheless, samples of one origin (wild-type or ∆ES7L_a_) also clustered, yet to a lesser degree, implying rather subtle differences between wild-type and ∆ES7L_a_ samples. Performing differential expression analysis, a list of proteins that significantly changed their association to ribosomes was obtained. GO term enrichment analysis of these proteins highlighted the “halfmer” formation since the only relevant GO term that was found to be significantly enriched was “translation initiation factor binding” (GO ID:0031369). However, when individual proteins were investigated, three aminoacyl-tRNA synthetases (MetRS, GlnRS and PheRS) with reduced binding to ribosomes lacking ES7L_a_ were found (Figure 3F). Importantly in WCL their levels were not changed suggesting a redistribution from ribosomes to the cytosol upon ES7L_a_ deletion (Figure 3G). AARS ribosome association does not appear to be stochiometric since the intensities measured in MS were lower in polysome associated fraction as compared to WCL. Immunodetection confirmed the substochiometric binding to ribosomes as the majority of signal was detected in slowly eluting fractions, i.e., fractions not containing polysomes (Figure 3H). Nevertheless, in wild-type lysates MetRS has increased association to polysomal fractions, which served as input material for mass spectrometry, as compared to ∆ES7L_a_ lysates as indicated in the first four lanes of the blot in Figure 3H. Furthermore, the migration behavior in later fractions, not containing polysomes, differed for wild-type and ∆ES7L_a_ cells. Whereas in the wild-type samples band intensities increased gradually, ∆ES7L_a_ samples display a bimodal distribution. Since these fractions did not contain polysomes and thus were beyond the scope of this study, this finding was not further investigated. While all synthetases could be tagged in the wild-type strain without any phenotypic effect, PheRS could not be tagged in ∆ES7L_a_ cells and cells expressing HA-tagged GlnRS displayed reduced fitness (Figure 3I). The combination of removal of ES7L_a_ and tagged PheRS and GlnRS appears detrimental for cells indicating that ES7L_a_ and synthetase association to the ribosome might contribute to cellular fitness. Furthermore, the order of magnitude by which the three synthetases were reduced in polysome fractions varied (Figure 3F), suggesting that ES7L_a_ is not equally relevant for binding of the three AARS. A role in AARS recruitment had previously been described for full length ES7L in vitro [13]. 

### 3.3. Aminoacyl-tRNA Synthetases Maintain Activity when Dissociated from the Ribosome

**Figure 4:**
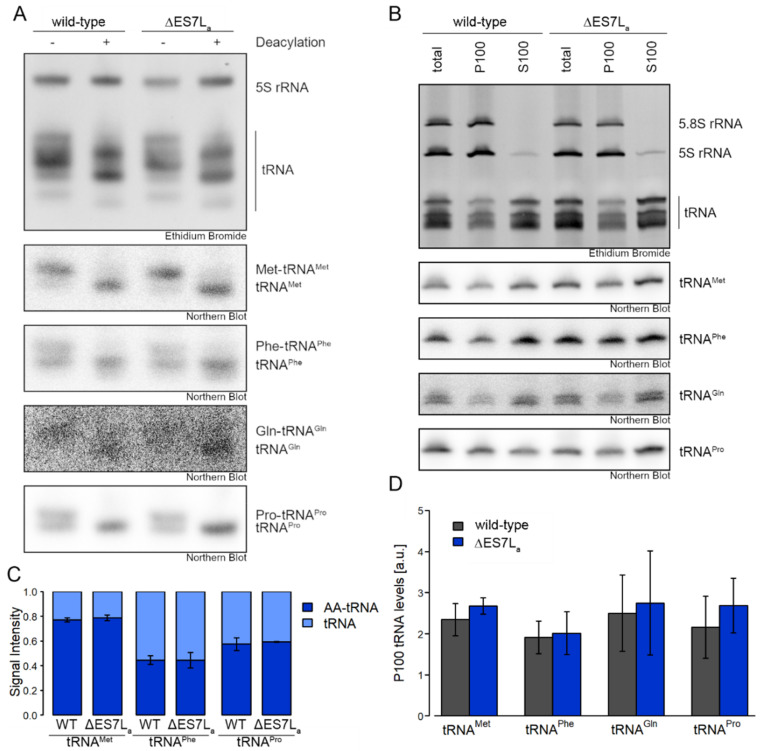
Acidic gel and P100/S100 northern blots. (**A**) Separation of aminoacylated and deacylated tRNA was performed by acidic polyacrylamide gel electrophoresis. Four tRNAs were detected by northern blot. For each RNA sample a deacylated control was included. (**B**) Quantification of signal intensities of the charged and uncharged tRNA in A (three biological replicates). (**C**) WCL was fractionated to ribosome-enriched pellets (P100) and the corresponding supernatant (S100) by centrifugation and tRNAs detected by northern blot. (**D**) Quantification of signal intensities of P100 tRNA levels normalized to 5S rRNA levels in the ethidium bromide staining in (**C**) (three biological replicates).

Early studies on AARS suggested more efficient aminoacylation of tRNAs by ribosome associated AARS [15]. If that hypothesis holds true, loss of ribosome association of the AARS as observed here, should reduce tRNA charging capacity. To address this question, aminoacylation levels were assessed by acidic northern blots for tRNA^Phe^, tRNA^Met^, tRNA^Gln^ and tRNA^Pro^ as a control (Figure 4A,B). In vivo-derived charged and uncharged tRNAs were sufficiently separated in all cases. None of the tRNAs, however, displayed altered aminoacylation levels between wild-type and ∆ES7L_a_ strains, suggesting that total tRNA charging does not depend on ribosome association (Figure 4A,B). To ensure that tRNAs are equally used in translation in both strains, we isolated P100, a crude ribosome-enriched pellet fraction, and measured tRNA levels associated to ribosomes (Figure 4C,D). Again, no difference in ribosome association of tRNAs was observed for all tRNA species tested, suggesting that tRNAs are fully charged and thus used in translation. These results suggest that despite delocalization to the cytosol, AARS do not loose charging activity and furthermore that tRNAs are equally loaded on to ribosomes during translation.

## 4. Discussion

### 4.1. ∆ES7L_a_ rRNA Maturation Is Perturbed and Translation Efficiency Reduced

Previous deletion studies on ES7L, the largest ES in yeast, showed, that its complete removal is lethal [5,34]. For that reason, and to obtain insights into ribosome biology beyond maturation here only the A-arm of ES7L was deleted. Deletion caused a rather mild phenotype evidenced by slower growth, reduced translation rates and altered biogenesis (Figure 1 and Figure 2). Many ES have been described to play a role in ribosome biogenesis and can be categorized into three classes, early middle and late acting ES [5]; ES7L is an early acting ES with a role in the nucleolus [5]. Our data suggests that A-arm deletion perturbs maturation in the nucleolus by reducing release from the nucleolus but barely by degrading the rRNA. As a result, we detect higher amounts of 27S precursor but only slightly reduced levels of large ribosomal subunits. This data agrees with the “halfmer” phenotype seen in polysome profiles (Figure 2C and Figure 3D); reduced levels of 60S subunits cause delayed subunit joining at the start codon which finally results in reduced overall translation as seen in metabolic labelling experiments. A similar scenario was observed in a strain lacking the eukaryote specific expansion of uL4, a ribosomal protein directly contacting ES7L [35]. This data strengthens the idea of a directed coevolution of eukaryote specific ES and ribosomal proteins, which form interaction networks at the ribosomal surface [5].

### 4.2. Aminoacyl-tRNA Synthetase Recruitment Is No Prerequisite for tRNA Charging

Having a clean and robust system at hand that allows the expression of a pure ribosome population harboring a deletion of a particular rRNA expansion segment enables us to test a longstanding hypothesis in the field of translation. In particular we were interested in testing whether or not local aminoacyl-tRNA charging on the ribosome occurs and if so if it contributes to translational competence. Several aminoacyl-tRNA synthetases (AARSs) have been reported previously to associate to ribosomes in different organisms and tissues [36,37,38]. These proteins directly influence protein biosynthesis by providing the substrate (aminoacylated tRNAs) for translation. In archaea, AARS bind the ribosome and directly recharge tRNAs after translation and channel them towards elongation factor 1 [39]. To which extend such mechanisms act in eukaryotes, such as yeast, is not fully understood. Yet, studies suggest that AARS bind translation complexes via the mRNA in mammals, suggesting a rather passive role of ribosomes in recruitment [20]. Yeast ES7L, however, was shown to bind AARS in vitro [13]. While the B-arm of ES7L is tightly bound by ribosomal proteins, the A-arm is flexible and extends towards the cytosol [29]. Based on these structural and *in vitro* features of ES7L we hypothesized ES7L to be a recruitment platform for cotranslationally acting factors and more specifically AARS. Indeed, we found several AARS to bind actively translating ribosomes (Figure 3F). Among them, three AARS showed reduced association to ribosomes when ES7L_a_ was deleted suggesting a recruitment role of this ES. While we saw reduction in ribosome binding, we observed, that the major fraction of AARS is actually not bound by ribosomes and thus only few wild-type ribosomes associate to these AARS. Whether AARS recruit to ribosomes translating particular mRNA transcripts with a high demand for specific amino acids remains elusive. In recent years, the idea of specialized ribosomes (i.e., ribosomes with a special molecular composition, enabling specific translational tasks) was proposed, which endows the ribosome with a higher regulatory potential in development and differentiation than previously thought [40,41,42,43]. Association of AARS to translating ribosomes and thus channeling of tRNAs which results in higher local rates of codon-specific translation and potentially altered cotranslational folding pathways would be a new mechanism to spatially control protein folding and function [15,44].

In our experimental data, the reduction of ribosome-association as evident in the ES7L_a_ deletion strain, however, did not change AARS aminoacylation activity on a global scale, since the corresponding tRNAs were charged equally well in wild-type and ∆ES7L_a_ cells. A similar trend has recently been reported for AARS associating to the MSC [24]. While loss of MSC association did not alter tRNA charging or ribosomal speed, the subcellular localization of AARS was altered, suggesting that the MSC serves as a regulator of subcellular AARS levels. Similar roles can be envisioned for AARS binding to ribosomes. To which extend, however, their localization is modulated by ribosome association remains to be investigated.

## Data Availability

The mass spectrometry proteomics data have been deposited to the ProteomeXchange Consortium via the PRIDE [45] partner repository with the dataset identifier PXD029751.
